# Competition Nutrition Practices of Elite Male Professional Rugby Union Players

**DOI:** 10.3390/ijerph18105398

**Published:** 2021-05-18

**Authors:** Logan Posthumus, Kirsty Fairbairn, Katrina Darry, Matthew Driller, Paul Winwood, Nicholas Gill

**Affiliations:** 1 Faculty of Health, Education and Environment, Toi Ohomai Institute of Technology, Tauranga 3112, New Zealand; paul.winwood@toiohomai.ac.nz; 2 Te Huataki Waiora School of Health, The University of Waikato, Hamilton 3216, New Zealand; nicholas.gill@nzrugby.co.nz; 3 New Zealand Rugby, Wellington 6011, New Zealand; kirsty@fuelmypotential.com (K.F.); katrina.darry@nzrugby.co.nz (K.D.); 4 School of Allied Health, Human Services and Sport, Sport and Exercise Science, La Trobe University, Melbourne 3086, Australia; m.driller@latrobe.edu.au; 5 Faculty of Applied Science, Auckland University of Technology, Auckland 0627, New Zealand

**Keywords:** dietary analysis, game day, macronutrients, energy intake, fuelling, team-sport

## Abstract

Thirty-four elite male professional rugby union players from the New Zealand Super Rugby championship completed dietary intakes via the Snap-N-Send method during a seven-day competition week. Mean seven-day absolute energy intake was significantly higher for forwards (4606 ± 719 kcal·day^−1^) compared to backs (3761 ± 618 kcal·day^−1^; *p* < 0.01; *d* = 1.26). Forwards demonstrated significantly higher mean seven-day absolute macronutrient intakes compared to backs (*p* < 0.03; *d* = 0.86–1.58), but no significant differences were observed for mean seven-day relative carbohydrate (3.5 ± 0.8 vs. 3.7 ± 0.7 g·kg·day^−1^), protein (2.5 ± 0.4 vs. 2.4 ± 0.5 g·kg·day^−1^), and fat (1.8 ± 0.4 vs. 1.8 ± 0.5 g·kg·day^−1^) intakes. Both forwards and backs reported their highest energy (5223 ± 864 vs. 4694 ± 784 kcal·day^−1^) and carbohydrate (4.4 ± 1.2 vs. 5.1 ± 1.0 g·kg·day^−1^) intakes on game day, with ≈62% of total calories being consumed prior to kick-off. Mean pre-game meal composition for all players was 1.4 ± 0.5 g·kg^−1^ carbohydrate, 0.8 ± 0.2 g·kg^−1^ protein, and 0.5 ± 0.2 g·kg^−1^ fat. Players fell short of daily sports nutrition guidelines for carbohydrate and appeared to “eat to intensity” by increasing or decreasing energy and carbohydrate intake based on the training load. Despite recommendations and continued education, many rugby players select what would be considered a “lower” carbohydrate intake. Although these intakes appear adequate to be a professional RU player, further research is required to determine optimal dietary intakes.

## 1. Introduction

Rugby union (RU) is a high-contact, field-based team sport contested by two teams over an 80-min game which is divided into two 40-min halves separated by a break no longer than 15-min [1]. Each team has 15 players on the field at a time comprising eight forwards (numbers 1–8) and seven backs (numbers 9–15), with each positional group having their own unique physical and fitness characteristics [2] and game-play demands [1]. Game-play encompasses both high-intensity (sprinting, tackling, lifting, rucking, mauling, scrumming) and low-intensity activities (standing, walking, jogging). Players typically cover distances of ≈5–7 km during a game with an average heart rate of ≈172 bpm [3,4] and are involved in numerous high-impact collisions (≈0.4 per minute) depending on playing position [5,6]. Meanwhile, distances of ≈8–10 km and a total weekly internal training load of ≈1500–1800 arbitrary units (AU) from all training sessions during an in-season competition week have previously been reported among professional RU players [7].

Professional rugby players are exposed to considerable training loads in season, resulting in substantial energy outputs [7,8,9], glycogen utilisation [10,11], exercise-induced muscle damage from training and game-play, and extensive impact-induced muscle damage, particularly from collisions during game-play [5,6,12,13,14,15]. In order to optimally prepare, fuel, and recover from the in-season training and game-play demands within professional RU, players must consume adequate amounts of food in order to meet energy and macronutrient requirements to promote muscle regeneration, glycogen restoration, and immune support, as well as to reduce fatigue [16].

Currently, studies among professional RU players have assessed dietary intakes during pre-season [17,18] and in-season [7] periods. During the in-season, mean energy intakes for forwards (≈3966 kcal·day^−1^) and backs (≈3392 kcal·day^−1^) have been observed alongside what would be considered a “relatively low” carbohydrate (≈3.5 g·kg·day^−1^), high-protein (≈2.7 g·kg·day^−1^), and moderately high fat (≈1.5 g·kg·day^−1^) macronutrient intake [7] compared to team-sport nutrition recommendations (carbohydrate = ≈5–7; protein = ≈1.6; fat = ≈1.3 g·kg·day^−1^) [16,19,20] and general sport nutrition recommendations for carbohydrate on intense training and competition days (6–10 g·kg·day^−1^) [21,22].

Although dietary intakes have been observed in season [7], no data have been published regarding the dietary intakes of professional RU players on game day (GD). Assessing dietary intake on GD will help provide further understanding of the energy and macronutrient intakes during an entire competition week and will demonstrate how GD compares to training and rest days during the week. Moreover, no data are currently available on how dietary intake is distributed prior to and following a game in professional RU. It is also important to note that no in-season dietary intakes have been reported within southern hemisphere professional RU players. Being able to compare in-season dietary intakes between northern [7] and southern hemisphere professional RU players would provide valuable insights regarding global nutritional approaches for RU [23].

Therefore, the aim of this study was to investigate the in-season dietary intakes of elite male professional RU players during a seven-day competition week. Dietary intake on GD was captured to determine how GD compares to other days during the competition week and how energy and macronutrient intake is distributed pre- and post-game. It was hypothesised that forwards would have a significantly higher mean seven-day energy and macronutrient intake compared to backs and that both positions would consume significantly more energy and macronutrient intakes on GD compared to training and rest days during the week. The quantification of field, gym training loads, and game loads were calculated and presented to help provide context for the observed dietary intakes.

## 2. Materials and Methods

### 2.1. Study Design

A cross-sectional observational study design was utilised to assess the in-season dietary intakes of elite male professional RU players during a competition week including GD. Seven-day remote photographic food diaries (Snap-N-Send method) with two-way communication to confirm measures and descriptions were recorded. Dietary intake across the competition week was catalogued as days away from GD (GD-5, GD-4, GD-3, GD-2, GD-1, GD, GD+1), as previously reported [7,8]. It is important to note that GD-3 and GD+1 were rest days (Table 1). Kick-off on GD was ≈7:00 p.m., which was preceded by a pre-game eating window from first food consumed that day right up until kick-off, with a pre-game meal consumed 3–4 h prior to kick-off. The post-game eating window commenced immediately following the game (≈9:00 p.m.) until players went to sleep. All players within this study competed on GD.

### 2.2. Participants

Thirty-four elite male professional RU players (age: 27.6 ± 2.8 y, height: 187.5 ± 8.5 cm, body mass: 103.0 ± 13.6 kg) from New Zealand Super Rugby Championship teams participated in this study. Players were categorised by their primary playing position, which comprised 17 forwards (27.8 ± 2.4 y, 193.4 ± 6.7 cm, 114.3 ± 6.8 kg) and 17 backs (27.5 ± 3.3 y, 181.5 ± 5.3 cm, 91.8 ± 8.2 kg). Mean years of professional playing experience and sum of eight-site skinfolds for all players were 8.5 ± 2.8 y and 61.0 ± 13.2 mm, respectively. All participants provided informed consent and the research was approved by the University of Waikato Human Research Ethics Committee (HREC 2019#04).

### 2.3. Anthropometrics

Following methods previously described [2], we assessed body mass upon waking with bladder voided at the start of the competition week using electronic scales (SECA, Birmingham, UK) to 0.1 kg accuracy. Height was assessed using a stadiometer (SECA, Birmingham, UK) to 0.5 cm accuracy. A level 1 International Society for the Advancement of Kinanthropometry (ISAK) accredited anthropometrist carried out the sum of eight-site skinfold measurements on all players using Harpenden callipers (British Indicators, Hertfordshire, UK) to 0.1 mm accuracy. Duplicate measures were taken on the right side of the body at the following sites: triceps, subscapular, biceps, iliac crest, supraspinale, abdominal, mid-thigh, and medial calf [24]. All anthropometric equipment were calibrated as recommended by the manufacturer’s guidelines.

### 2.4. Training Load

Quantification of field, gym, and game internal loads were assessed using sRPE [25], wherein the participants’ rated each session on the basis of intensity using a modified 10-point Borg Scale [26] which was then multiplied by the total training or game time in minutes (RPE × total training time = sRPE), providing the sRPE in the form of an AU. Session values were then summed for each individual to provide a daily and seven-day total for internal training load (Table 1). This method has previously been used in professional RU [7,17] and rugby league (RL) [8,27,28]. External training load from field sessions and game-play were collected for each player via 10-Hz global positioning system (GPS) units (VX Sport, Wellington, New Zealand) and software (VX Sport, Wellington, New Zealand) providing daily and seven-day total distance (Table 1).

### 2.5. Dietary Intake Assessment and Analysis

Dietary intake was assessed using seven-day remote photographic food diaries recently referred to as the “Snap-N-Send” method [29,30,31,32]. The “Snap-N-Send” method is a dietary assessment tool that has been validated for use within elite adolescent rugby athletes and has demonstrated enhanced validity (mean bias for under-reporting = ≈4% vs. ≈14%) and reliability (typical error of estimates = ≈5% vs. ≈11%) over traditional dietary assessment tools [29] and may provide greater compliance while reducing burden on players [30,33]. The smartphone application MealLogger^®^ (version 4.7.4, Wellness Foundry, Helsinki, Finland) was used to photograph and record what participants consumed [34]. Players provided a brief description of what foods were in each photograph, outlining brand names; cooking methods; ingredient amounts; and items difficult to quantify and identify within the photo such as oils, sauces, and condiments. Players were encouraged to measure out food amounts whenever possible. As part of their regular sports nutrition servicing to enhance their food literacy, all players had received prior education regarding the quantification of the foods they were consuming in grams or in cups.

Once meal entries were received, the picture and description were checked to ensure suitability for analysis. Any images or entries received that required further clarification were followed up via MealLogger messaging where players provided further details as required. For quality inputs to be ensured, all players received training to teach them how to use the software and practice the process prior to data collection. Dietary intakes were captured, entered, and analysed by a single registered sports dietitian with extensive experience in analysing photograph food diaries and working with RU players. To enhance the accuracy of the information collected [35], we provided the resulting seven-day food record to the players’ sports nutritionist to enable in-person validation with the player for any errors in representation of foods and beverages consumed. Any corrections were returned to the sports dietitian analyst who then adjusted the record accordingly. All meals were entered into the dietary analysis software FoodWorks 10 Professional (Xyris, Australia).

### 2.6. Statisical Analyses

All statistical tests were analysed using the Statistical Package for the Social Sciences (SPSS, v25, IBM, New York, NY, USA). All data were checked for normality using the Shapiro–Wilk test (*p* > 0.05). Differences between forwards and backs for mean seven-day and pre-game meal energy and macronutrient intakes were assessed using independent *t*-tests. Repeated measures analysis of variance (ANOVA) using pairwise comparisons with a Bonferroni adjustment was used to analyse differences in energy and macronutrient intake on GD compared to the remaining days of the competition week among forwards and backs. Mauchly’s test of sphericity was assessed and accounted for using methods previously described [7]. Differences between pre- and post-game energy and macronutrient intakes for forwards and backs were analysed using paired samples *t*-tests. An alpha level of *p* ≤ 0.05 was used for all tests. Effect sizes were calculated using the Cohen’s *d* method with the following thresholds; *d* = *trivial* < 0.19, *small* 0.20–0.49, *medium* 0.50–0.79, and *large* > 0.80 [36]. All data are expressed as mean ± standard deviation.

## 3. Results

### 3.1. Seven-Day Energy and Macronutrient Intake

Mean seven-day energy and macronutrient intakes among forwards and backs are presented in Table 2. Forwards demonstrated significantly higher mean seven-day absolute energy (*p* < 0.01), carbohydrate (*p* = 0.02), protein (*p* < 0.01), and fat (*p* = 0.03) intakes compared to backs. No significant differences were observed for mean seven-day relative energy and relative macronutrient intakes between positions.

### 3.2. Daily Energy Intake

**Figure 1:**
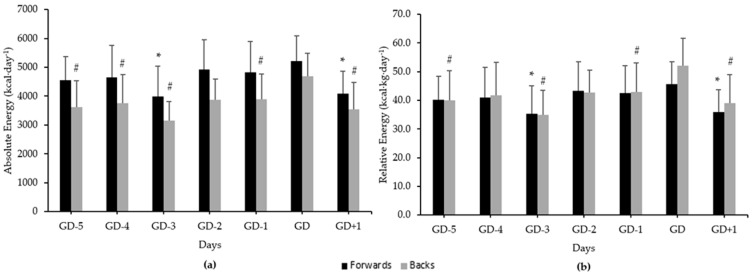
Mean daily energy intakes across a seven-day competition week (GD-5, -4, -3, -2, -1, GD, GD+1) among forwards and backs. (**a**) Absolute energy intake; (**b**) relative energy intake. Error bars represent standard deviation. * Indicates a significant difference to GD for forwards (*p* < 0.05). # Indicates a significant difference to GD for backs (*p* < 0.05).

Mean daily energy intake among forwards and backs can be observed in Figure 1. There was a significant difference between days for absolute (*F*(6, 96) = 6.81, *p* < 0.01) and relative (*F*(6, 96) = 6.65, *p* < 0.01) energy intakes among forwards. Significantly higher absolute and relative energy intakes were observed on GD compared to GD-3 (*p* < 0.01; *d* = 0.90–0.85, respectively) and GD+1 (*p* < 0.01; *d* = 0.98–0.90, respectively) among forwards. There was a significant difference between days for absolute and relative energy intakes among backs (*F*(6, 96) = 9.42, *p* < 0.01). Backs demonstrated significantly higher absolute energy intake on GD compared to GD-5 (*p* < 0.01; *d* = 0.90), GD-4 (*p* < 0.05; *d* = 0.74), GD-3 (*p* < 0.01; *d* = 1.51), GD-1 (p < 0.01; *d* = 0.68), and GD+1 (*p* < 0.01; *d* = 0.95). Relative energy intake among backs was significantly higher on GD compared to GD-5 (*p* < 0.01; *d* = 0.87), GD-3 (*p* < 0.01; *d* = 1.35), GD-1 (*p* = 0.01; *d* = 0.66), and GD+1 (*p* < 0.01; *d* = 0.96).

### 3.3. Daily Macronutrient Intake

**Figure 2:**
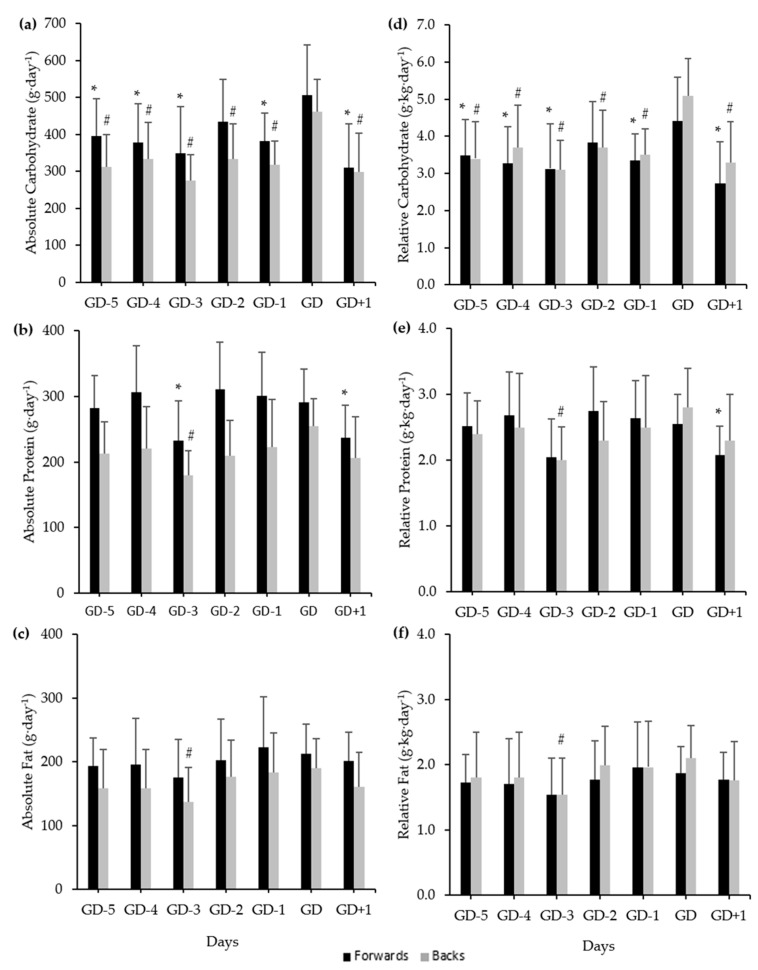
Mean daily macronutrient intake across a seven-day competition week (GD-5, -4, -3, -2, -1, GD, GD+1) among forwards and backs. (**a**) Absolute carbohydrate intake; (**b**) absolute protein intake; (**c**) absolute fat intake; (**d**) relative carbohydrate intake; (**e**) relative protein intake; (**f**) relative fat intake. Error bars represent standard deviation. * Indicates a significant difference to GD for forwards (*p* < 0.05). # Indicates a significant difference to GD for backs (*p* < 0.05).

Mean daily macronutrient intakes among forwards and backs can be observed in Figure 2. There was a significant difference between days for absolute (*F*(6, 96) = 8.55, *p* < 0.01) and relative (*F*(6, 96) = 8.26, *p* < 0.01) carbohydrate intakes among forwards. Significantly higher absolute and relative carbohydrate intakes on GD compared to GD-5 (*p* = 0.03; *d* = 0.65–0.60, respectively), GD-4 (*p* = 0.02; *d* = 0.74–0.70, respectively), GD-3 (*p* = 0.02; *d* = 0.84–0.77, respectively), GD-1 (*p* = 0.01; *d* = 0.79–0.72, respectively), and GD+1 (*p* < 0.01; *d* = 1.08–1.04, respectively) were observed among forwards. There was a significant difference between days for absolute (*F*(6, 96) = 7.29, *p* < 0.01) and relative (*F*(6, 96) = 7.22, *p* < 0.01) protein intakes among forwards. Absolute protein intake was significantly higher on GD compared to GD-3 (*p* = 0.04; *d* = 0.73) and GD+1 (*p* = 0.02; *d* = 0.75) among forwards, but relative protein intake on GD was only significantly higher than GD+1 (*p* = 0.03; *d* = 0.71). Backs demonstrated significant differences between days for absolute (*F*(6, 96) = 12.53, *p* < 0.01) and relative (*F*(6, 96) = 12.75, *p* < 0.01) carbohydrate intakes, with significantly higher absolute and relative carbohydrate intakes on GD compared to all other days (*p* < 0.01; *d* = 0.96–1.65). There was a significant difference between days for absolute (*F*(6, 96) = 4.50, *p* < 0.01) and relative (*F*(6, 96) = 4.50, *p* < 0.01) protein intakes among backs, with significantly higher absolute and relative protein (*p* < 0.01; *d* = 1.33–1.15, respectively) intakes observed on GD compared to GD-3. Backs also demonstrated significant differences between days for absolute (*F*(6, 96) = 3.23, *p* = 0.01) and relative (*F*(6, 96) = 3.00, *p* = 0.01) fat intake, with significantly higher absolute and relative fat (*p* = 0.03; *d* = 0.75–0.77, respectively) intakes observed on GD compared to GD-3.

### 3.4. Pre- and Post-Game Energy and Macronutrient Intake

Mean energy and macronutrient intakes for pre- and post-game among forwards and backs are presented in Table 3. Both forwards and backs demonstrated significantly higher absolute and relative energy and macronutrient intakes pre-game compared to post-game (*p* < 0.01).

### 3.5. Pre-Game Meal Energy and Macronutrient Intake

**Figure 3:**
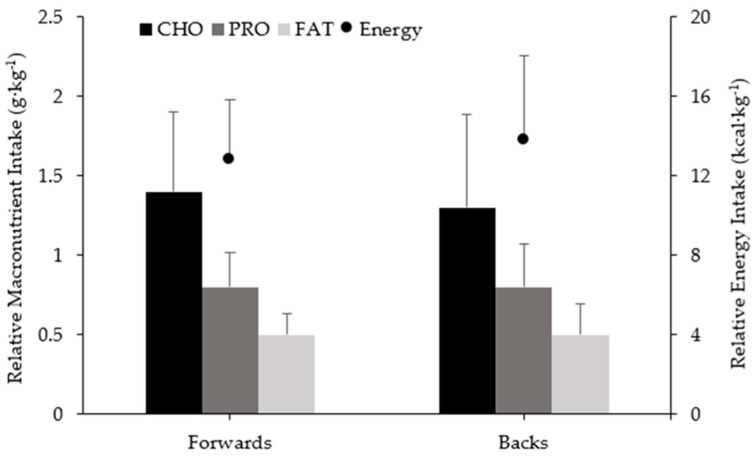
Relative energy and macronutrient intakes of pre-game meal 3–4 h prior to kick off among forwards and backs. CHO = carbohydrate, PRO = protein, FAT = fat. Error bars represent standard deviation. No significant differences observed (*p* > 0.05).

Mean relative energy and relative macronutrient intakes for the pre-game meal within forwards and backs can be observed in Figure 3. No significant differences in energy or macronutrient intake were present for the pre-game meal between positions.

## 4. Discussion

This study is the first to report the dietary intakes of elite male professional RU players on GD, including pre-game, pre-game meal, and post-game dietary intakes and how GD compares to other days during the competition week. Within this study, forwards consumed significantly higher mean seven-day absolute energy and absolute macronutrient intakes compared to backs. Interestingly, relative to bodyweight, no significant differences were observed between positions regarding seven-day mean relative energy and relative macronutrient intakes. In fact, backs demonstrated higher mean seven-day relative energy and relative carbohydrate intakes compared to forwards. Significantly higher absolute and relative energy intakes were observed on GD compared to rest days and numerous training days among backs, but only compared to rest days for forwards. However, both positions demonstrated significantly higher relative and absolute carbohydrate intakes on GD compared to all training (except GD-2 for forwards) and rest days. Both forwards and backs consumed significantly higher absolute and relative energy and macronutrient intakes pre-game compared to post-game, with no significant difference in pre-game meals between positions.

Our study reported higher mean absolute energy intakes for forwards (4606 ± 719 vs. 3966 ± 299 kcal·day^−1^) and backs (3761 ± 618 vs. 3392 ± 287 kcal·day^−1^) compared to a previous study measuring dietary intakes within professional RU players during the in-season competition period [7]. When comparing macronutrient intakes within our study to those reported by Bradley et al. [7], we observed identical mean relative carbohydrate intakes among forwards (3.5 ± 0.8 vs. 3.5 ± 0.8 g·kg·day^−1^), while backs consumed slightly more carbohydrate in our study (3.7 ± 0.7 vs. 3.4 ± 0.7 g.kg·day^−1^). Mean relative protein intake was slightly lower within our study for both forwards (2.5 ± 0.4 vs. 2.7 ± 0.5 g·kg·day^−1^) and backs (2.4 ± 0.5 vs. 2.7 ± 0.3 g·kg·day^−1^), while mean relative fat intake was higher within our study for both forwards (1.8 ± 0.4 vs. 1.4 ± 0.2 g·kg·day^−1^) and backs (1.8 ± 0.5 vs. 1.4 ± 0.3 g·kg·day^−1^) compared to Bradley et al. [7]. In comparison to dietary intakes reported during the pre-season period among professional RU players, mean absolute energy intakes were higher within our in-season study, yet relative macronutrient intakes were similar regardless of seasonal period [17,18].

Despite differences in game-play [1] and physical and fitness characteristics [2] between positions, forwards and backs had similar dietary intakes relative to bodyweight, although forwards demonstrated significantly higher absolute dietary intake. Similar to previous studies in professional RU [37], players were found to be consuming a relatively low carbohydrate (≈3.6 g·kg·day^−1^), high protein (≈2.5 g·kg·day^−1^), and moderate–high fat (≈1.8 g·kg·day^−1^) intake compared to recommendations for team sport athletes [16,19,20,37]. Although a range of players within this study are meeting the mean intake of ≈3.5 g·kg·day^−1^ or greater, there are still players who are not meeting these values, which highlights the range of individual differences and the potential continued education required to ensure appropriate fuelling is occurring. Furthermore, the lower relative carbohydrate intakes within professional RU players could partly be due to the large body mass of these players, as discussed in a recent review [23].

The protein intakes observed in our study seem appropriate for professional RU players given their training and match demands that generate high levels of exercise-induced and impact-induced muscle damage [15]. These intakes may also be suitable for players who need to lose unwanted fat mass while maintaining or gaining lean muscle mass [38,39,40,41,42]. These higher protein intakes may also be contributing to the relatively high fat intakes observed, although players also attempt to select foods containing healthy fats. Due to the caloric density of dietary fat, these relatively high fat intakes may be beneficial in helping meet energy requirements of professional RU players [20] due to the large body mass [2] and high energy expenditures reported [8,9].

Mean daily absolute energy intake followed a similar trend to previously reported in-season data [7] where players consumed slightly less energy at the start of the week, which then increased closer to GD. The higher mean energy intakes observed within our study may partly have been due to the greater total distances covered (≈17 km) and higher total sRPE (≈3670 AU) reported from training sessions alone, which are more than double the training loads observed by Bradley et al. [7] and are also greater than loads previously reported during the pre-season period [17]. These perceived internal training loads followed the same trend as daily dietary intake across the competition week, which indicate that the RU players observed within this study “eat to intensity”. As perceived internal training load (sRPE) increases and decreases, energy and macronutrient (particularly carbohydrate) intake follows the same trend to “fuel for the work required” [43]. However, “eating to intensity” or increasing dietary intake only when exercise intensity increases does not necessarily mean optimal fuelling is occurring, as there may be lower-intensity days when increasing dietary intake is desired to optimise fuel stores such as the day before a game (GD-1) [19]. While periodising carbohydrate in this manner may increase glycogen adaptations [44], it is likely players are selecting this eating pattern to fuel performance while attempting to control body composition [7,17].

Compared to northern hemisphere players, carbohydrate intake was higher on GD-5 within our study but similar across the rest of the days except GD-1, which was considerably higher for both forwards and backs in northern hemisphere players [7]. Other than GD, players within our study prioritised high energy and carbohydrate intake on GD-2, which is logical due to this day having the highest training load for the week, but then decreased energy and most notably carbohydrate intake on GD-1. Although logical from an intensity perspective, as GD-1 has the lowest training load for the week other than rest days (GD-3 and GD+1), from a fuelling perspective, due to GD being the following day, higher carbohydrate intake may be optimal [19].

Current recommendations for athletes are to consume between 6 and 10 g·kg·day^−1^ of carbohydrate, particularly around GD-1 in order to optimise glycogen stores [19,21]. However, mean carbohydrate intakes of ≈3.4 g·kg·day^−1^ on GD-1 were observed within our group, with the highest intakes recorded for a forward and back on GD-1 being ≈4.7 g·kg·day^−1^. In contrast, RU players in the northern hemisphere [7] were closer to the lower end of these recommendations [21] with considerably higher mean carbohydrate intakes on GD-1 for forwards (≈5.1 g·kg·day^−1^) and backs (≈4.2 g·kg·day^−1^) compared to our study. Unfortunately, Bradley et al. [7] were unable to measure dietary intake on GD, which would have been interesting to observe whether GD carbohydrate and energy intakes were higher than on GD-1 as reported within our study.

The carbohydrate amounts observed within this study may be adequate to replenish glycogen stores. Bradley et al. [10] reported no significant differences in pre-match, half-time, and post-match glycogen stores within RL players who consumed 3 or 6 g·kg·day^−1^ of carbohydrate 36 h prior to an 80-min game. Although glycogen stores appeared more homogenous within the 6 g·kg·day^−1^ group, no clear differences were observed in GPS and workload metrics between the two groups [10]. However, similar research within RU players is required to provide further insight regarding glycogen utilisation. Interestingly, these “lower” carbohydrate intakes (≈3.5 g·kg·day^−1^) have supported improvements in physical performance and body composition measures during pre-season phases composed of high-intensity and high-volume glycogen depleting training sessions [17,18], suggesting these intakes may be adequate.

On GD, it has been suggested that athletes may eat less and therefore consume less carbohydrate due to game stress, potential travel, and match schedules. Thus, increased fuelling on GD-1 has been suggested to offset this potential decrease in dietary intake on GD [19]. This was not the case within the players we studied, with GD demonstrating higher dietary intake compared to the rest of the week. Later start times (≈7:00 p.m. kick-off) are likely a large contributor to these higher intakes, allowing players adequate time to fuel prior to the game, demonstrated by players consuming ≈62% of their total energy intake for the day during that time. The pre-game meal (3–4 h prior to kick-off) consisted of approximately 1.4 g·kg^−1^ carbohydrate, 0.8 g·kg^−1^ protein, and 0.5 g·kg^−1^ of fat for both positions, meeting suggested pre-match fuelling requirements for carbohydrate [21].

Within the ≈3 h eating window post-game, players were near the suggested refuelling requirements with ≈1–1.2 g·kg^−1^ of carbohydrate consumed immediately after the game followed by another ≈0.5–1 g·kg^−1^ of carbohydrate an hour later to optimise glycogen replenishment, which may have been further enhanced by the amount of protein ingested (≈1.1 g·kg^−1^) by the players during this period [16,19]. The absolute carbohydrate intakes observed post-game within this study were similar to the amounts (180 g) and timings (immediately and 1 h post-game) suggested to promote glycogen replenishment in RL players [11]. Immediate re-feed strategies may be crucial during these night-time games due to the limited post-game eating window, with players still attempting to get to sleep at a reasonable hour following the game. On GD+1, energy intake was higher than a normal day off during the middle of the week (GD-3), which may help offset the higher resting metabolic rates observed post-game due to the physical demands and collisions experienced during game-play [13,14].

A limitation to this study was that no measure of energy expenditure was implemented to determine energy requirements and daily energy balance. Given the large internal and external training loads observed, future research should examine the energy expenditures of these elite professional RU players. Another limitation is that we were unable to measure muscle glycogen stores alongside dietary intakes, which would better inform the adequacy of carbohydrate consumption across the competition week. It is also important to note that assessing dietary intake is subject to error by both the analyst and the participant [29,32,33,35]. Therefore, there may be some error in our dietary data due to under- or over-reporting, even though we put processes in place to mitigate errors as much as possible.

## 5. Conclusions

The dietary intakes observed within this study are similar to those previously reported in male professional RU players, demonstrating relatively low carbohydrate, high protein, and moderate–high fat intakes compared to sports nutrition recommendations. Although these RU players appear to meet fuelling requirements in the pre-game meal and post-game eating window, they do not meet the suggested daily carbohydrate fuelling requirements. This information is useful for practitioners working with RU players, given that successful and experienced elite professional RU players have been studied and appear to perform adequately with these dietary intakes observed given their selection in elite professional teams. However, to ensure “optimal” rather than “adequate” fuelling is being achieved, we require more studies examining energy balance and carbohydrate intake alongside changes in glycogen stores and performance measures within professional RU players. This will help guide practitioners as to whether (a) players are meeting energy requirements; (b) more education and strategies around helping RU players increase carbohydrate intake to meet recommendations are required; or (c) the recommendations are too high for this population group, and therefore the “relatively lower” carbohydrate intakes reported in the literature are sufficient given the large body mass and physical demands of rugby players.

## Figures and Tables

**Table 1:** Seven-day overview, training, and game load during the competition week.

	GD-5	GD-4	GD-3	GD-2	GD-1	GD	GD+1	Total
**All Players**			Rest and Recovery				Rest and Recovery	
Intensity	Low	High		High	Low	High		
Field sessions	1	1		2	1	0		5
Gym sessions	1	1		1	0	0		3
sRPE (AU)	784 ± 123	1277 ± 108		1367 ± 90	242 ± 42	716 ± 94		4386 ± 457
Distance (km)	2.4 ± 1.0	6.7 ± 0.8		5.1 ± 0.7	2.7 ± 0.5	6.1 ± 0.7		23.0 ± 3.7
**Forwards**								
sRPE (AU)	781 ± 118	1266 ± 114		1377 ± 97	238 ± 41	695 ± 99		4357 ± 469
Distance (km)	2.1 ± 0.8	5.7 ± 0.7		4.4 ± 0.7	2.5 ± 0.4	5.5 ± 0.8		20.2 ± 3.4
**Backs**								
sRPE (AU)	788 ± 132	1289 ± 104		1356 ± 84	247 ± 44	737 ± 86		4416 ± 449
Distance (km)	2.8 ± 1.2	7.6 ± 0.9		5.7 ± 0.7	2.9 ± 0.5	6.7 ± 0.6		25.7 ± 3.9

Mean ± standard deviation. GD = game day (GD-5–GD-1 refers to days prior to GD, GD+1 refers to day following game), sRPE = session rate of perceived exertion, AU = arbitrary unit. Low intensity = mean day RPE < 6, high intensity = mean day RPE > 6.

**Table 2:** Seven-day mean energy and macronutrient intakes among forwards and backs.

Dietary Intake		Forwards (*n* = 17)	Backs (*n* = 17)	Effect Size
Energy	kcal·day^−1^	4606 ± 719 (3500–6132) *	3761 ± 618 (2465–4978)	1.26
	kcal·kg·day^−1^	40.5 ± 7.2 (28.4–56.3)	41.9 ± 7.2 (29.8–53.8)	0.19
Carbohydrate	g·day^−1^	399 ± 77 (268–525) *	340 ± 59 (174–415)	0.86
	g·kg·day^−1^	3.5 ± 0.8 (2.5–4.8)	3.7 ± 0.7 (2.1–4.6)	0.27
	% TEI	35 ± 5 (28–44)	36 ± 5 (30–48)	0.20
Protein	g·day^−1^	280 ± 39 (227–372) *	220 ± 37 (155–302)	1.58
	g·kg·day^−1^	2.5 ± 0.4 (1.8–3.4)	2.4 ± 0.5 (1.7–3.3)	0.22
	% TEI	24 ± 2 (21–28)	23 ± 3 (19–29)	0.39
Fat	g·day^−1^	210 ± 43 (144–288) *	169 ± 41 (103–242)	0.98
	g·kg·day^−1^	1.8 ± 0.4 (1.1–2.5)	1.8 ± 0.5 (1.1–2.6)	0.00
	% TEI	41 ± 4 (33–45)	41 ± 5 (28–47)	0.00

Mean ± standard deviation (range). % TEI = percentage of total energy intake. * Indicates a significant difference between forwards and backs (*p* < 0.05).

**Table 3:** Energy and macronutrient intakes pre-game (first meal of day to last meal prior to kick-off) and post-game within forwards and backs.

Dietary Intake		Forwards (*n* = 17)			Backs (*n* = 17)		
		Pre-Game	Post-Game	ES	Pre-Game	Post-Game	ES
Energy	kcal^−1^	3363 ± 759 ^†^	1860 ± 601	1.55	2786 ± 496 ^‡^	1908 ± 661	1.06
	kcal·kg^−1^	29.4 ± 6.5 ^†^	16.3 ± 5.5	1.54	30.8 ± 5.6 ^‡^	21.3 ± 7.9	0.98
Carbohydrate	g^−1^	339 ± 102 ^†^	167 ± 84	1.30	271 ± 55 ^‡^	190 ± 74	0.88
	g·kg^−1^	3.0 ± 0.9 ^†^	1.5 ± 0.7	1.32	3.0 ± 0.5 ^‡^	2.1 ± 0.8	0.95
Protein	g^−1^	185 ± 57 ^†^	106 ± 26	1.26	144 ± 27 ^‡^	111 ± 35	0.75
	g·kg^−1^	1.6 ± 0.5 ^†^	0.9 ± 0.2	1.30	1.6 ± 0.3 ^‡^	1.2 ± 0.4	0.80
Fat	g^−1^	132 ± 36 ^†^	81 ± 36	1.00	119 ± 35 ^‡^	71 ± 31	1.03
	g·kg^−1^	1.2 ± 0.3 ^†^	0.7 ± 0.3	1.18	1.3 ± 0.4 ^‡^	0.8 ± 0.4	0.88

Mean ± standard deviation. ES = effect size. ^†^ Indicates a significant difference to post-game among forwards (*p* < 0.01). ^‡^ Indicates a significant difference to post-game among backs (*p* < 0.01).

## Data Availability

The data are not publicly available due to privacy. All data is presented clearly and honestly.
